# White Sharks (*Carcharodon carcharias*) Scavenging on Whales and Its Potential Role in Further Shaping the Ecology of an Apex Predator

**DOI:** 10.1371/journal.pone.0060797

**Published:** 2013-04-09

**Authors:** Chris Fallows, Austin J. Gallagher, Neil Hammerschlag

**Affiliations:** 1 Apex Expeditions, Cape Town, South Africa; 2 Leonard and Jayne Abess Center for Ecosystem Science and Policy, University of Miami, Coral Gables, Florida, United States of America; 3 RJ Dunlap Marine Conservation Program, University of Miami, Miami, Florida, United States of America; 4 Rosenstiel School of Marine and Atmospheric Science, University of Miami, Miami, Florida, United States of America; University of Toronto, Canada

## Abstract

Scavenging, a result of a temporary pulse of resources, occurs in virtually all ecosystems containing carnivores, and is an important energy transfer pathway that can impact ecosystem structure and function, and this ecological significance has largely been considered from a terrestrial standpoint; however, little is known about the role of scavenging in shaping the behavioral ecology of marine species, specifically apex predators. Here we present findings from multiple opportunistic observations of white sharks scavenging on whale carcasses in False Bay, South Africa. Observations of white sharks scavenging over successive days provided evidence of strategic and selective scavenging by this species. Moreover, extended daily observations permitted recordings of unique social, aggregative, and feeding behaviors. We further compare these data against observations of natural predation by sharks on seals in the study area. We discuss these data in relation to environmental conditions, shark social interactions, migration patterns, whale biology, and behaviorally-mediated trophic cascades. While the appearance of a whale carcass is largely a stochastic event, we propose that white shark scavenging on whales may represent an underestimated, yet significant component to the overall foraging ecology of this species, especially as individuals attain sexual maturity.

## Introduction

Scavenging, a result of a temporary pulse of resources, is a type of multi-channel feeding [1], [2] that facilitates both bottom-up and top-down regulation of populations through different trophic levels [3], [4]. In vertebrates, scavenging represents an important energy transfer pathway in many ecosystems, and can be induced in various ways such as predator kills and animal death due to disease and malnutrition, whereby the deceased becomes carrion.

**Figure 1:**
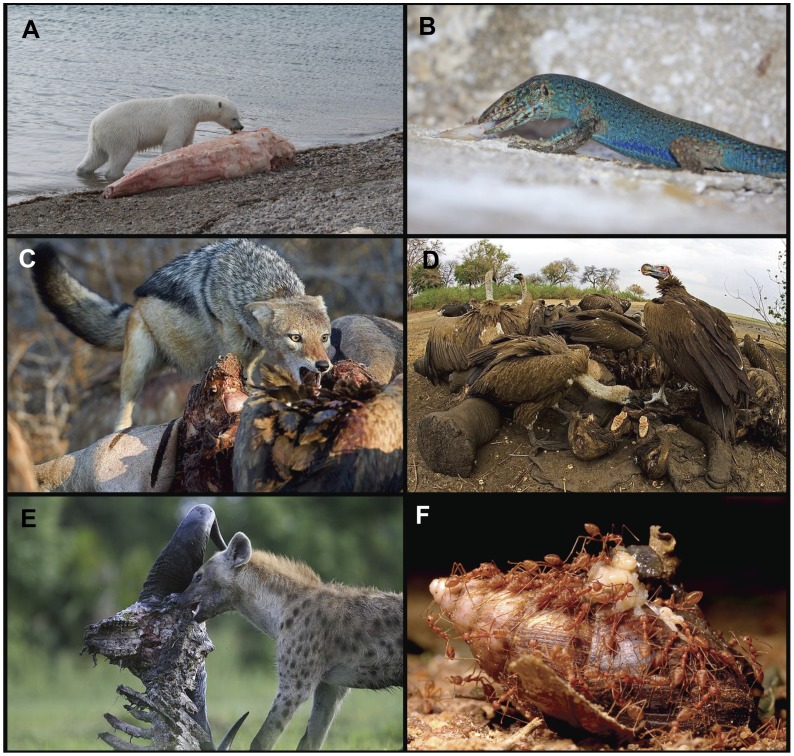
Scavenging occurs in virtually all food-webs and habitats. (A) a polar bear (*Ursus maritimus*) eating flesh from a narwhal whale carcass (*Monodon monoceros*) (Photo: Jeff W. Higdon/DFO); (B) an Ibiza wall lizard (*Podarcis pityusensis*) scavenging on fish scraps leftover from another predator (Photo: Nate Dappen/Day’s Edge Productions); (C) a black backed jackal (*Canis mesomelas*) scavenges on a zebra *(Equus quagga)* kill (Photo: Chris Fallows); (D) lappet faced vulture (*Torgos tracheliotos*) and white backed vultures (*Gyps africanus*) scavenge on an elephant kill (Photo: Chris Fallows); (E) A spotted hyena (*Crocuta crocuta*) removes flesh from a long-dead ungulate (Photo: Chris Fallows); (F) red weaver ants (*Oecophylla smaragdina*) gathering to feed on a dead African giant snail (*Achatina fulica*) (Photo:Narasha Mharte).

Carrion availability and quality ranges across spatiotemporal scales in virtually all food webs, and as such, multi-species scavenger guilds can become nested within carnivore (and omnivore) trophic levels in many ecosystems [5](Figure 1). A dead or decaying carcass of an animal essentially creates a new habitat or resource patch in which consumers target and exploit through scavenging. Such a sudden input of energy (i.e., carrion/carcasses) creates a resource subsidy that lowers the energetic costs of consumption a predator would incur otherwise via hunting or predation, which in turn can create a fiercely competitive de-coupling of food-web dynamics [5]. For example, a single dead ungulate carcass in a terrestrial ecosystem (i.e., forest or savannah) can induce scavenging across multiple trophic level consumers such as apex predatory mammals (e.g., bears, wolves, lions), secondary mammalian consumers (e.g., small carnivorous rodents, foxes, hyenas), as well as air-borne consumers (e.g., eagles, hawks, vultures, insects). Furthermore, scavenging can comprise an important component of a predator’s ecology (i.e., facultative scavenger) [6]. Spotted hyena (*Crocuta crocuta*), for example, are generally regarded as among the most efficient predators in the sub-Saharan Africa due to the combination of their strong hunting skills as well as their highly successful opportunistic scavenging capabilities [7], [8].

Terrestrial studies suggest that scavenging could be more frequent in ecologically complex food-webs [9]; however, the inclusion of scavenging in trophic models has generally been overlooked [10]. Documenting scavenging events is inherently challenging due to the stochastic nature of the events, a pattern that is especially difficult in marine systems given the logistical constraints of surveying the vast expanse of the ocean, as well as the concealing nature of the environment.

In marine systems, the largest source of carrion is that of a whale carcass [11]. Consequently, the role of dead whale carcasses in promoting ecosystem function has been studied via bottom-up processes involving benthic invertebrate and microbial communities (i.e., “whale falls”) [11]–[13]. However, very little is known about the impact such an immense input of organic matter may have on the ecology of predatory fishes through scavenging, a knowledge gap that is likely driven by the fact that many whales perish and sink in remote areas of the oceans, where frequent researcher observation is limited.

The white shark (*Carcharodon carcharias*) is the largest extant predatory fish, growing over 6 m in length [14]. Based on stomach content and stable isotope analyses, white sharks are believed to exhibit ontogenetic diet shifts, expanding their diet from primarily foraging on fish and elasmobranchs as juveniles to also including marine mammal prey, such as cetaceans, as adults [15]–[18]. However, observations of white sharks feeding on cetaceans, including scavenging, are extremely rare worldwide. Dating as far back as 1896, there have only been 19 published accounts in the primary literature of white sharks scavenging on whale carcasses [19]–[22]. Of these, only two studies [19], [20] analyzed the behavior of white sharks scavenging at the carcass, with the former being the only to describe multiple (2) individuals simultaneously feeding from the same whale, although intra-specific interactions were not observed. Given the stochastic nature, yet potentially strong signal of a decaying whale (an abundant, energy-rich, food source), congregations of otherwise solitary, and rare, apex predators scavenging on a whale carcass creates the potential for initiating changes in trophic interactions and ecological processes through top-down forcings.

Here we present data analyzing multiple white shark scavenging events on baleen whales in False Bay, South Africa. We also compare these data with observations of natural predatory strikes by white sharks on Cape Fur seals (*Arctocephalus pusillus pusillus*) at this site [23], [24]. We use these insights to further our understanding of cetacean scavenging by these apex predators and propose that it may be an important aspect of the overall foraging ecology of the white shark across its ontogeny. This added dimension of predatory tactics is discussed as it relates to environmental conditions, shark social behavior, migration patterns, whale biology, and behaviorally-mediated trophic cascades.

## Materials and Methods

Seal Island, South Africa (S 34.1374; E 18.5825), is an elongated rocky islet at the foot of False Bay, inhabited by approximately 60,000 Cape Fur seals. During winter months (∼May – September), white sharks actively patrol the waters around the Island for seals leaving and returning from foraging offshore [23].

Observations of white sharks scavenging events occurred opportunistically at Seal Island as part of our ongoing long-term ecological study (1997 to present day) on predator-prey interactions between white sharks and Cape Fur seals that occur at the site. These study methods are described in detail in [23] and [24]. Briefly, during the study period, our team surveyed the waters around Seal Island between 7∶00 hr to 1330 hrs for both seal activity and predation events. When a predatory attack by a shark on a seal was detected, we recorded (where possible) the total length (TL) of the attacking shark (estimated to the nearest 0.5 m against known dimensions of the boat), seal age class, direction of seal movement (towards or away from the Island), number of seals in the group attacked and whether the seal was consumed by the shark or if it escaped (see [23] and [24]).

On four occasions throughout our ongoing study, we had the opportunity to observe white shark scavenging on whale carcasses (see below and Table 1). When a scavenging event occurred, we anchored our boat alongside the carcass to document white shark scavenging behavior as well as consumption of the carcass (Table 1). At each event, we recorded the species and size of whale as well as tried to infer cause of death. At the start of each daily observation period, the following environmental conditions were recorded: (1) water visibility; (2) swell height; (3) water temperature; (4) wind speed and (5) wind direction.

**Table 1:** White sharks scavenging from two species of whales based on four separate accounts in False Bay, South Africa.

Whale #	Date	Whale Species	Size (m)	Wind Dir.	Wind Spd. (knots)	Temp	Obseration Duration (hrs)	Max # sharks	Min Size (m)	Max Size (m)
1	05-Jul-00	*Balaenoptera edeni*	11	SE	7.5	14.5	5	40	2.7	5
1	06-Jul-00	*Balaenoptera edeni*	11	LV	1	14.3	5	10	2.2	4.5
2	15-Aug-02	*Eubalaena australis*	10	SE	15	13.5	5	20	2.2	4.5
2	16-Aug-02	*Eubalaena australis*	10	SE	17.5	13.5	13	5	3.3	5
2	17-Aug-02	*Eubalaena australis*	10	SE	25	13.5	3	2	4.4	5.5
2	20-Aug-02	*Eubalaena australis*	10	LV	1	13.5	7	2	4.2	5
2	21-Aug-02	*Eubalaena australis*	10	NE	11	13.5	6	10	3.4	4.8
2	22-Aug-02	*Eubalaena australis*	10	NE	11	13.5	6	4	3.5	4.5
3	07-Sep-03	*Eubalaena australis*	18	NW	27.5	15.5	3	4	3.8	4.3
4	09-Sep-10	*Balaenoptera edeni*	11	LV	1	14.4	3	1	3	3
4	10-Sep-10	*Balaenoptera edeni*	11	SE	12	14.1	12	15	2.4	4.3
4	11-Sep-10	*Balaenoptera edeni*	11	LV	1	14	10	8	2.3	3.8
4	12-Sep-10	*Balaenoptera edeni*	11	S	6	14.3	6	6	3.2	4.2
4	13-Sep-10	*Balaenoptera edeni*	11	LV	1	14.3	8	3	3.6	3.7
4	14-Sep-10	*Balaenoptera edeni*	11	S	4	14.7	5	1	3.1	3.1

For each shark observed within 10 m of the carcass, we recorded, where possible, the shark TL (following [24]); shark sex (based on presence or absence of claspers) and the behavior of any shark within the vicinity of the carcass.

At the time, one of our team (CF) had tagged over 70 individual sharks with color-coded tags such the majority of sharks could be identified at the individual level. The remainder of sharks could be identified at the individual level based on unique body markings (following [25]) and dorsal fin morphology (following [26]) made from above and below water.

Following the approach of Curtis et al. [19], we similarly defined a feeding bout as the presence of a shark at the surface near the carcass and consisting of at least one attempt to remove flesh. In addition to recording the size, sex and behavior of the shark, when a feeding bout occurred, we also noted the time of the event and location on the whale where the scavenging bout occurred. Similar data were also recorded if any intra-specific interactions were observed. Throughout each day, we also continued to record seal movement and predatory activity by sharks using the approach of Hammerschlag et al. [24] and Martin et al. [27].

Assessment of shark occurrence (using the count of the maximum number of sharks observed) during scavenging forays was conducted for Whale #2 and Whale #4, as these instances afforded sufficient multi-day observation for analyses. Due to the nature in which shark occurrence was quantified (counts per day), as well the shape of its distribution, an exploratory Poisson regression was used to investigate the potential influence of various operational and environmental variables on white shark occurrence at the carcass; the independent explanatory variables investigated were: days the carcass was floating, whale type (species 1 or 2), sea surface temperature (°C), observational effort (hours/day), and wind speed (knots). Generalized linear models were constructed with backwards-forwards stepwise selection, starting with a model containing all explanatory variables. Retention or removal of variables were based on Akaike Information Criterion (AIC), with the lowest AIC suggesting the best fitting model. Given that wind speed is a continuous variable, the influence wind speed on maximum shark size observed at the carcass (TL) was investigated via linear regression.

To examine the relationship between predatory attacks on seals and the concurrent whale scavenging events, we compared daily rates of shark predations on seals during the days when the whale carcass was present (termed “During”) versus those occurring over a two week period before and after (termed “Before” and “After”). These data were not collected for whale #3 and thus not analyzed. Data collected for whale #4 only included (and analyzed for) predation events occurring before and during scavenging events. Since predation data (daily counts of predatory attacks on seals) did not conform to the assumptions of normality and homogeneity of variance, data from all whales were grouped and we used Kruskal-Wallis tests to compare daily shark predations rates on seals before, during and after the occurrence of the whale carcass at Seal Island. Statistical significance was declared at p<0.05, and all analyses were performed in the R statistical program (R Development Core Team 2009) except for the predation data, which were analyzed using SAS (SAS Institute, Cary, North Carolina, USA).

### Whale One (5–6 July 2000)

On 5 July 2000, an adult male Bryde's whale (*Balaenoptera edeni*), was found dead at Glencairn, in False Bay, South Africa (S 34.1634, E 18.4326). The cause of death to the 11 m animal was due to massive injury to the lower jaw, suspected to have occurred as a result of a collision with a boat. To dispose of the carcass, rather than using explosives to break up the carcass to smaller pieces and then bury, the South African Navy decided to tow the carcass to Seal Island where they knew it would be scavenged and consumed by sharks (Table 1). The fresh Bryde’s whale carcass was anchored by the Navy 100 m off the eastern side of the Island at approximately 1300 hrs. Wind conditions were southeast at approximately 5–10 knots. Once the whale was secured, we anchored our vessel next to the whale and began observations and data collection. Observations began at 1300 hrs and were curtailed at 1900 hrs. On 6 July 2000, data were recorded continuously between 0820 hrs and 1330 hrs.

### Whale Two (15–22 August 2002)

On 15 August 2002, a male southern right whale (*Eubalaena australis*), was found dead at Millers point, in False Bay (S 34.2287, E 18.4724). The cause of death to the 10 m animal was unknown. To dispose of the carcass as previously done, it was towed by the South African Navy to Seal Island (Table 1). The whale carcass was anchored by the Navy off the northern side of the Island at approximately 1400 hrs. Wind conditions were southeast at 15 knots and water temperature was measured at 13.5°C. The whale appeared to be in a highly decayed state. Once the whale was secured, we anchored our vessel next to the whale and began observations and data collection. We left the whale at approximately 1900 hrs. On 16 August, the wind speed and direction was southeast 15–20 knots, with water temperature at 13.5°C. Scavenging observations occurred between 0715 hrs and approximately 1800 hrs. On 17 August, observations occurred from 0815 hrs until 1100 hrs due to bad weather, with winds blowing 25 knots out of the southeast. On 20 August, winds were light and variable at the Island, allowing us to anchor at the whale at 0700 hrs. Observations were made until 1400 hrs. On both 21 and 22 August, observations were made between approximately 0800 hrs and 1400 hrs. Wind speeds hovered around 10–12 knots out of the northeast. Water temperature was 13.5°C.

### Whale Three (15 September 2003)

On 15 September 2003, an adult southern right whale was found drifting 3 km from Seal Island (Table 1). The ∼16 m whale appeared to be very fresh and four sharks were already feeding on the carcass on our arrival. The cause of death was unknown. Observations only lasted three hours, because we were forced to return to port due to strong northwest winds (25–30 knots). When the research team was absent, the winds were believed to have pushed the carcass out the bay and offshore.

### Whale Four (9–14 September 2010)

On 9 September 2010, a dead 11 m Bryde’s whale was found drifting 1.6 km east of Partridge point, just South of Millers Point in False Bay. When the whale was found, it had a large commercial fishing rope entangled around its fluke, suggesting that cause of death was likely a result of an interaction with a commercial fishing operation. Our team towed the carcass to Seal Island at 1315 hrs (Table 1). Wind was light and variable and water temperature was measured at 14.4°C. The carcass was anchored at 1423 hrs about 200 m off the northern side of the Island. Observations were recorded until 1715 hr, after which we departed the carcass. On 10 September, we arrived at the carcass at 0635 hrs. Wind direction and speed were southeast 10–12 knots and water temperature 14.1°C. Data were recorded until 1700 hrs. On 11 September, we arrived at the whale carcass at 0702 hrs. During the observational period, the wind was light and variable and water temperature was measured at 14.0°C. We monitored shark scavenging on the whale carcass until 1710 hrs. On 12 September, we arrived at the carcass at 652 hr. The wind direction and speed was south at 6 knots; water temperature was 14.3°C. At 1305 hrs we needed to leave the carcass due to strong winds. On 13 September, wind conditions were light and variable, permitting us to monitor the whale for shark scavenging from 0654 until 1437 hrs. On 14 September, we arrived at the whale carcass at 0723 hrs. The wind was out of the south at 3–5 knots; the water temperature was 14.7°C. At about 1200 hrs, the wind strengthened, severed the anchoring line and the carcass drifted out of sight.

## Results and Discussion

Previous authors have hypothesized that whale carcasses probably account for a large portion of adult white shark’s diet, as scavenging events may become important to their foraging along migration routes [21], [28]–[30]. To date, our understanding of white shark scavenging behavior on whale carcasses comes from less than twenty observations. Of these, detailed analyses were based on two single instances reported by Curtis et al. [19] and Dicken [20]. In the former, analysis was based on three white sharks scavenging on a humpback whale (*Megaptera novaeangliae*), where no obvious intra-specific interactions among sharks were documented. The latter case reported on several juvenile sharks scavenging on a humpback whale in Angola Bay, South Africa; however, simultaneous feeding was not observed. In the present study, the number of different sharks observed scavenging on a given day ranged from 1 to 40 individuals, with over 50% of the observational days documenting more than 5 different sharks scavenging (Table 1).

**Figure 2:**
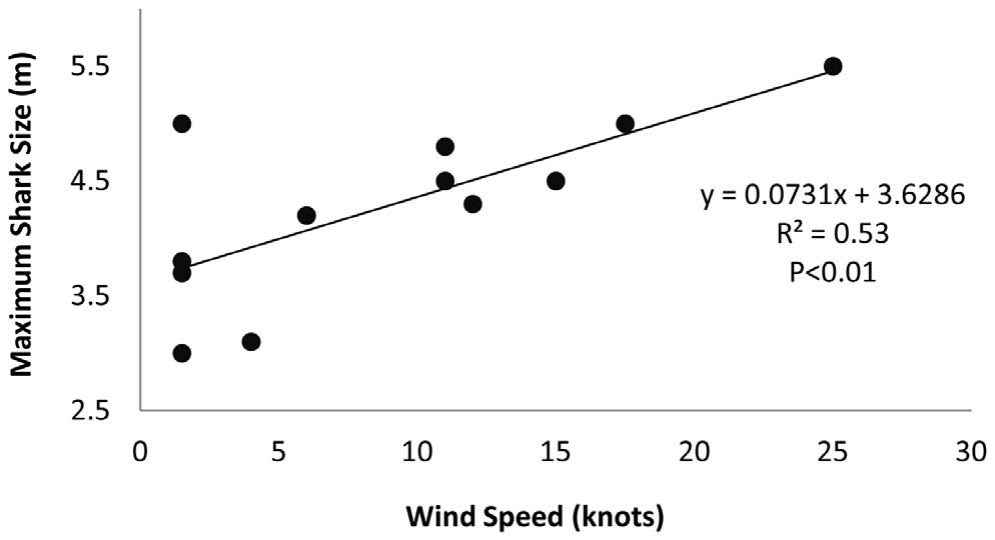
Regression of wind speed and maximum shark size observed at all scavenging events in the present study.

Quantitative investigations of scavenging events in the literature are relatively rare; however, we conducted a preliminary analysis to explore potential relationships between several explanatory variables (days the carcass was floating, observational effort, wind speed, temperature, whale type) and shark occurrence at the carcass. Diagnostics of the modeling procedures are shown in Table 2. The best exploratory model (Model 1) indicated that, after partitioning the variance associated with observational effort, significant effects were detected for days the carcass spent floating and the prevailing wind speed (Table 2). Results from this strongest exploratory Poisson regression model suggest that wind speed (knots) increased the likelihood of observing white sharks at the carcass (p<0.001, Table 3), while detecting an inverse relationship between days the carcass spent floating and shark occurrence (p<0.05, Table 3). In this final model, whale type (individual differences) and water temperature had no effect on white shark occurrence at the carcass. We also detected a significant positive linear relationship between the maximum size of sharks at the carcass (each day) and wind speed (R^2^ = 0.53, F_(1,11)_ = 11.11, p<0.01; Figure 2). Taken together, these data reveal two things. First, that the ability to detect increasing numbers of sharks during scavenging is partly based on researcher effort, a trend that suggests our knowledge and understanding of scavenging in marine predators is inherently impeded by limited documentation at large temporal scales. Second, that shark detection of the strong odor cues emanating from a whale carcass may be driven by environmental factors (discussed later).

**Table 2:** Model diagnostics for generalized linear models for Poisson distributed data modeling shark occurrence during whale carcass scavenging forays.

Model	Variables included in the model	AIC	ΔAIC	Significant Variables
1	Day, effort, windspeed	83.1	0	Day,* effort,** windspeed **
2	Day, effort, windspeed, SST	84.4	1.3	Day,* effort*
3	Day, effort, whale type	84.5	1.4	Day,** effort,** whale type*
4	Day, effort, windspeed, whaletype	84.9	1.8	Day,* effort*
5	Day, effort, SST, whale type, windspeed	85.5	2.4	wind*

Days the carcass spent floating (day), observational effort (effort) sea surface temperature (SST), whale carcass species (whale type), and windspeed (windspeed) were included in the models. The best 5 models are shown, all others had ΔAIC values >2.5. Predictor variables with significant (p<0.05) effects on shark occurrence are indicated with an asterisk.

* p<0.05;

** p<0.01;

*** p<0.001.

**Table 3:** Summary results of the best model the using Poisson regression in Table 2 to examine effects of biological and environmental variables on shark occurrence during scavenging forays on the two whale carcasses.

Parameter	Coefficient estimate	SE	z-value	*P*
Days Floating	−0.821	0.334	−2.460	0.014
Effort (hours/day)	0.747	0.260	2.873	0.004
Windspeed (knots)	0.318	0.103	3.093	0.002

**Figure 3:**
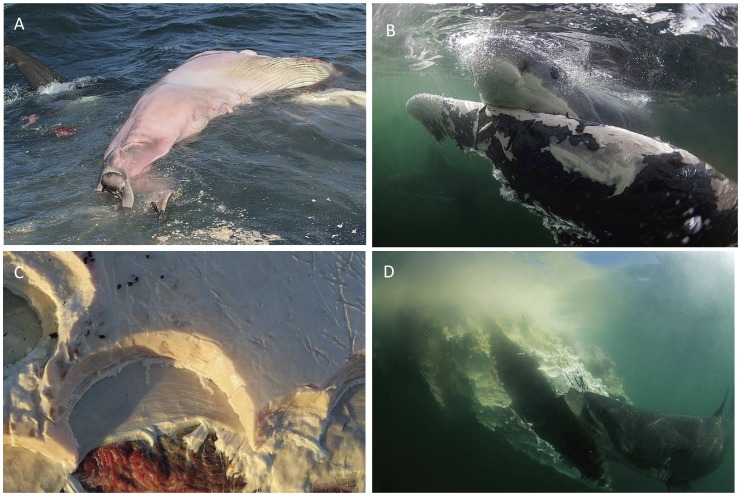
Examples of selective, facultative scavenging by white sharks on various whale carcasses in South Africa. (A) 4 m white shark removing and consuming a near-term fetus from a Bryde’s whale carcass; (B) white shark scavenging on caudal peduncle and fluke of a Bryde’s whale; (C) impression of a white shark bite on whale carcass through dermal, subcutaneous and blubber layer; (D) a white shark removing blubber around the jaw of a southern right whale carcass.

The relatively large number of sharks and long duration of time sharks were recorded scavenging during this study, including observations of the same individuals scavenging over successive days, permitted a unique opportunity to examine this aspect of white shark behavior (Table 1). For example, on 10 Sept 2010, while several sharks were simultaneously feeding on a Bryde’s whale, a 4 m shark moved into the carcass and removed out a near-term fetus from the whale’s uterus that it then consumed (Figure 3A). Such an event may provide evidence to support strategic scavenging by this species.

In terms foraging efficiency, Carey et al. [28] estimated that 30 kg of whale blubber could satisfy the basal metabolism of a 4.5 m white shark for 1.5 months. Further, Klimley [31] and Klimley et al. [32] proposed that white sharks preferentially consume blubber-rich, high-fat prey such as cetaceans to fuel their elevated metabolic needs. This hypotheses continues to be heavily cited as a driver of white shark feeding ecology, but has only been documented in a few instances [19], [33]–[35]. Hammerschlag et al [36] suggested that white sharks will likely not reject low quality foods; however, when food is non-limiting, sharks will be selective for high caloric and nutritious items.

**Figure 4:**
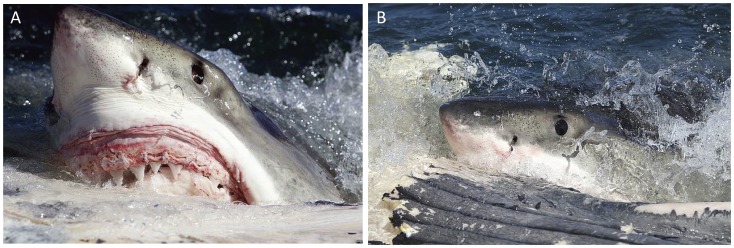
Examples of unique behaviors employed by white sharks during scavenging forays on whale carcasses in False Bay, South Africa. (A–B) A 4.5 m white shark removes a 20 kg chunk of flesh, sinew and blubber by performing lateral headshakes without employing protective ocular rotation.

In this study, sharks generally exhibited an initial preference for feeding on the whale caudal peduncle and fluke, before moving to feed along the rest of the body (Figure 3B). This was unexpected and has previously not been described. However, sharks also clearly showed preference for areas of high blubber content (Figure 3C–D), a finding which is consistent with previous studies and the hypotheses presented above [19], [20], [33]–[35]. Interestingly, individuals would typically approach the whale slowly, during which they would swim around and mouth different parts of the carcass, before settling on a specific blubber-rich spot and biting down repeatedly to remove flesh, a behavior suggestive of selectivity as previously proposed [31], [32]. Sharks would remove flesh by performing lateral headshakes; all without employing protective ocular rotation, whereby a single scavenging bout would typically last 15–20 seconds (Figure 4 A,B). Performance of the ocular rotation is a protective behavior, usually employed during attacking or feeding on prey to reduce potential eye injury from struggling prey. The lack of such a behavior during scavenging suggests that white sharks likely recognize the carcass as a non-mobile, non-threatening entity, although whether this ecological “knowledge” is innate or learned is unknown. Interestingly, we found that sharks would routinely regurgitate large chunks of whale blubber only to immediately return to the carcass and feed once again. Taken together, and given that white shark teeth appear to function as mechanoreceptors [36], we hypothesize that observed regurgitation-consumption behavior may represent sharks replacing low energy yield pieces with higher energy yield chunks.

Sharks continued to feed until it appeared they experienced what we believed to be post-porandial torpor. For example, over long feeding bouts (>6 hrs), shark would continue to feed to the point where they would cease lifting their heads above the waterline. Moreover, they would continuously bump the carcass in what appeared to be an attempt to feed, but despite mouthing the carcass, sharks were unable to achieve a sufficient bite to remove flesh, after which they would simply bounce off the carcass and slowly sink underwater. Overall patterns of feeding activity (numbers of sharks actively removing chunks of flesh) decreased following the initial day of highest scavenging activity, despite large pieces of the whale still being available to be scavenged upon. This suggests that, broadly, sharks eventually became satiated and/or lose interest when areas of the highest blubber content are stripped from the carcass.

Throughout most of our documentation, multiple sharks were usually scavenging on the carcass at the same time showing little to no intra-specific aggression. For example, on 5 July 2000 at 1640 hrs, 8 sharks were observed feeding simultaneously, frequently bumping into one another without obvious signs of aggregation among individuals. Furthermore, on July 5 at 1711 hrs, 7 sharks were simultaneously feeding on the whale. In this instance, two individuals were feeding 1.5 m apart from one another. As one of the sharks continued to remove blubber, it worked its way along the flank of the whale, subsequently biting the head of the neighboring shark, leaving 2 teeth embedded in the shark’s head. However, neither of the sharks appeared to be affected by this interaction, as both continued to feed along the whale’s flank without any response behavior observed. On the same carcass >3 large sharks up to 5.0 m in length were seen feeding belly-up next to each other at times with their pectoral fins overlapping.

A variety of evidence indicates that sharks are capable of various forms of social recognition and organization, including forming dominance hierarchies [37]. For example, studies have shown that smaller sharks will exhibit subordinate behaviors to larger individuals and vice versa [37]–[39]. In this study, although sharks did not display any overt aggression to one another; a clear size-based pecking order was found. The largest (>5 m) sharks showed dominance and spatial partitioning at the carcass, targeting areas of high blubber content; while small sharks fed predominantly on areas with less blubber, comprised of solid muscle. The smallest sharks (3–4 m) kept their distance from the whale, mostly scavenging on pieces of floating blubber that drifted away from the carcass.

In addition to dominance hierarchies, it worth considering that the presence of a whale carcass appeared to quickly attract large adult sharks that had not previously been observed by our research group in the area. Scavenging events around cetaceans might be the ideal situation for adult large sharks to meet before mating (R. Strong, pers comm.). In addition to sexually mature sharks congregating at a single food source, the state of stimulus-induced excitement could trigger mating (R. Strong, pers. Comm). Clearly, there is insufficient data to support this hypothesis, but we recommend that future investigations of white sharks scavenging on a whale carcass should look for potential signs of mating-related behavior [40].

White sharks are known to display a relatively high degree of dietary plasticity for an apex predator, derived from ontogenetic shifts in diet [41], [42], as well as their ability to detect and respond to short and long-term shifts in the spatial distribution of prey resources [43]. During the peak of the seal hunting season (May-August), the average shark size at Seal Island is 3.5 m, with sharks larger than 4 m being a relatively rare occurrence, representing only 11% of the observed population [44]. Over the course of 16 years, we have documented numerous individual sharks returning to hunt seals at Seal Island annually, but when they grow to be larger than 4 m, they seem to disappear altogether from the waters surrounding Seal Island [44]. However, during the accounts described here, in less than 24 hours, we observed over two dozen white sharks exceeding 4 m (and some over 5 m) feeding on the carcasses. Based on these observations, we speculate that when white sharks become too massive to actively out-maneuver highly agile Cape Fur seas [45], and/or cease to derive sufficient nutrition from seals, they stop actively hunting seals at Seal Island and alter their behavior to patrol False Bay of the offshore coastlines of South Africa for dead or weak cetaceans.

The large densities of cetaceans found year round in False Bay and throughout the greater Western Cape may provide an attractive food source for white sharks over a critical size. Recent data suggests that the populations of overwintering southern right whales, a protected species which shows high coastal affinity off South Africa, had been increasing at an annual instantaneous rate of ∼7% from 1969–1987, while populations continued to recover at the turn of the century [46]. While there has been recent evidence to support a causal link between an increasing marine mammal prey species and the frequency of attack by white sharks [43], further study is needed to prove such a correlation between sharks and cetaceans within our study area.

Interestingly, aerial surveys of Bryde’s whales have identified two separate and biologically distinct groups of whales inhabiting the waters of southern Africa: an inshore group (<20 miles) and an offshore group (>50 miles); [47]). Recent work suggests that the inshore population is on average smaller (mean size 10–12 meters) and more fecund (a 3-fold increase in annual ovulation rate) than their offshore counterparts [47], [48]. The east-west movements, smaller size, and higher ovulation rates of the inshore Bryde’s whale population may render them an easier and more detectable (i.e., mechanical handling and sensory perception of ovulatory cues) prey item for the populations of white sharks patrolling the southern coast of Africa. Furthermore, both of these whale species are known to come within <1 km of the coastline during the winter months (June – December), subjecting them to higher risks of boat collisions, stranding, and entanglement with fishing gear [49], [50]. The hypothesis that white sharks may track, harass, and prey on inshore whales is consistent with white shark tracking studies revealing a high degree of “on-and-offshore” patrolling movements parallel to the South African coastline [51], [52]. In free-ranging predators, random-walk Lévy movement has been attributed to searching for prey that are randomly or widely distributed, whereby consumers will alternate between periods of highly-tortuous movement and longer-distance, straight-line movement [53]. We hypothesize that when not hunting seals at rookeries, adult white sharks patrol the South African coastline using Lévy movements to encounter dead, dying or weak whales. Future shark tracking studies (using horizontal and vertical data) employing quantitative data analysis coupled with aerial surveys are an avenue for further research to test our hypothesis.

Although previous white shark-whale studies were not able to explore for potential impacts of environmental conditions on scavenging behavior, we found preliminary evidence that shark detection of the strong odor cues emanating from a whale carcass may be driven by environmental factors. Chemical as well as odor signal detection and transduction are high in marine systems, whereby the spatial gradient of odor plumes can rapidly expand and attract highly sensory consumers [54], [55]. In this study, when winds were light or were not blowing inshore, few sharks (in some cases none) were present at the carcass; however, when winds were strong and blew towards the coastline, we found many sharks (up to 28 individuals) at the carcass. For example, on Sept 9, 2010, the wind was predominantly west and no sharks were seen in the vicinity of the carcass. However, overnight the wind switched to southeast, such that the slick was heading toward the coastline and as soon as our boat arrived at the whale, there were already seven individual sharks simultaneously feeding on the whale. At the time of this feeding event, Seal Island had few white sharks present, with only 2 sharks being seen in the day’s preceding the whale carcass event. Of the sharks that were observed around the carcass, many were recognized as distinct individuals seen at Seal Island during the previous four months feeding on seals, but these sharks were absent in the weeks preceding the whale carcass (Authors, unpublished data). Taken together, these data suggest that when sharks are not present at the Island hunting seals, they may not entirely leave the confines of the region; instead, they may remain and forage within relative proximity from the bay as the maximum distance the whale carcasses slick could travel was 5 km from the Island under prevailing wind and current conditions. Further, the fact that increased wind speed brought larger sharks to the area (Figure 2), provides support for our hypothesis (as discussed above) that large, mature, sharks patrol the waters outside False Bay for dead and dying whales migrating through the region. When winds are strong enough such that the scent trail moves sufficiently offshore, large sharks may be drawn into the Bay to investigate and scavenge.

**Figure 5:**
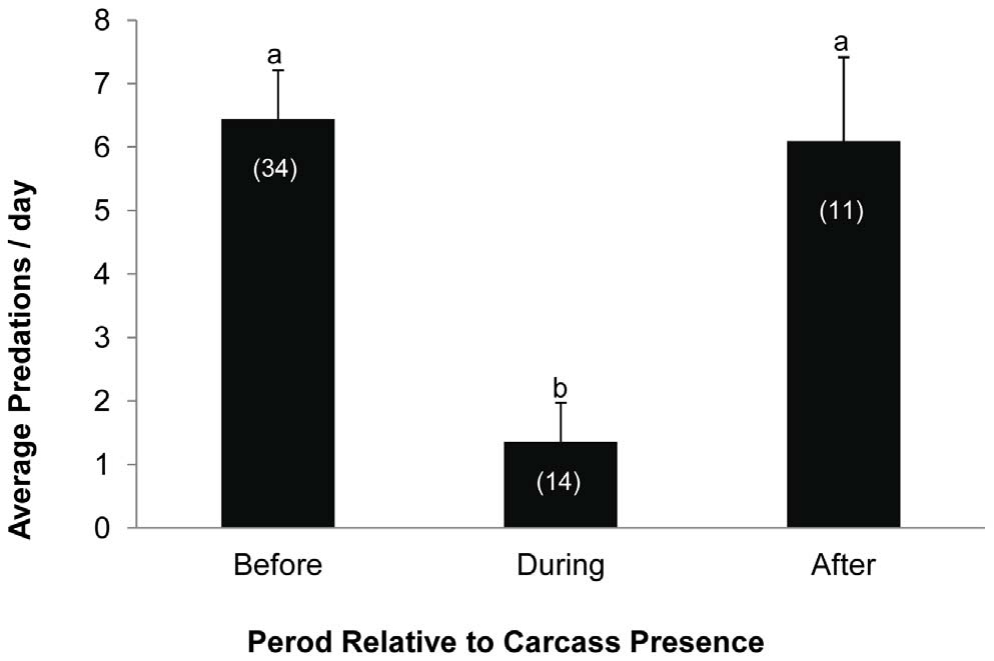
A comparison of mean shark predations on seals before, during and after the occurrence of a whale carcass at Seal Island. Data examined before and after were collected over a two week period. Data were pooled across whales (#1, #2, #4). Predation data (mean±SE) were not collected nor analyzed for the period following the occurrence of whale #4. Differences in lower case letters denote statistical differences (p<0.05). Numbers in parentheses indicate number of observational days. Error bars represent 1 standard error.

It is worth considering the potential for behaviorally mediated indirect interactions (BMIIs) arising from sharks scavenging on whales [56]. BMIIs occur when a change in an ‘‘initiator’’ species causes a behavioral shift in a ‘‘transmitter’’ species that, in turn, affects a ‘‘receiver’’ species, which can initiate trophic cascades [56]. By changing the foraging focus of white sharks (initiator), the whales (transmitter) indirectly influence the behavior, metabolism, and mortality on seals (receiver), and subsequently influence foraging of other secondary “receivers.” Indeed, we found that shark predation rates on seals were significantly lower during the periods when the carcass was present at the Island compared to the two weeks before or after (p<0.01); although there was no significant difference in predation rates before or after (P = 0.87, Figure 5). Such BMIIs have the capacity to alter the dynamics of the numerous species the seals and sharks interact with, including their prey. These indirect effects have been shown to have large effects in biologically rich ecosystems with multiple consumer guilds [57], [58].

**Figure 6:**
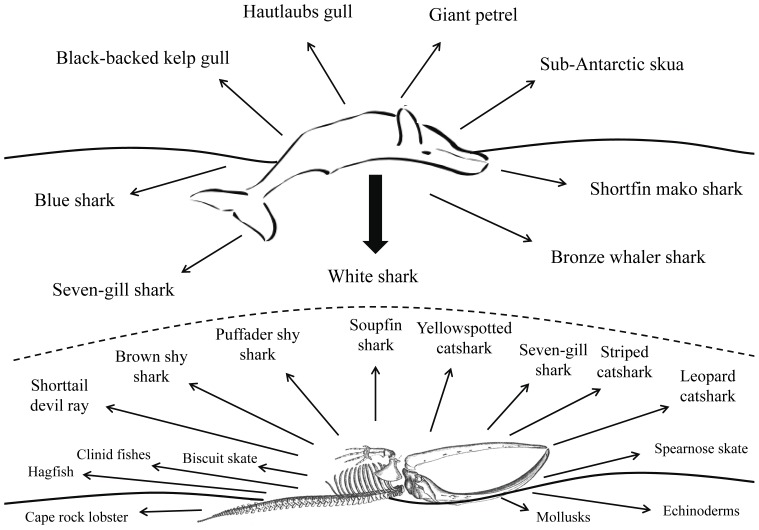
Activation and regulation of community-wide consumer food-web catalyzed via pulse of a whale carcass in False Bay, South Africa. Diagram showing range of scavengers on a whale carcass at the surface (above dotted line) and when the carcass sinks to the seafloor (bottom dotted line) following removal of blubber by white sharks.

The waters of False Bay, South Africa are supplied from the Agulhas and Benguela currents, and these productive oceanographic inputs are reflected in the ecological diversity and biomass of its marine faunal communities [59], [60]. Indeed, the roughly 906 km^2^ bay is home to over 5 species of marine birds, 15 species of elasmobranchs, various demersal and pelagic game fishes, thousands of dolphins from various species, 3 species of whales and over 64,000 cape fur seals (Authors direct observation); [24]. The pulse of a whale carcass in such a rich system provides an attractive and low-cost temporary resource for multiple species across the whale’s latent downward migration: mobile predatory fishes and avian opportunists on the surface, as well as smaller teleosts, cartilaginous fishes, benthic invertebrates and microbes once it has settled on the benthos (Figure 6). Through competitive exclusion and BMII’s, white sharks likely regulate the subsequent spatiotemporal availability for scavenging by other consumers on the carcass, while also suppressing or altering intra-specific foraging rates on other prey.

The ease of encountering and observing white sharks predating upon seals at seal rookeries worldwide likely creates a biased and misleading view of white shark foraging behavior [36]. Despite being efficient predators of Cape fur seals (48% success rate of capture at Seal Island) [24], we suggest that whale scavenging represents a critical and more frequent, but rarely observed, component of white shark ecology. Given that the stoichiometric quality of carrion is comparable to the tissue of scavengers and also because predators do not need to waste energy stalking, chasing and subduing prey, carrion is assimilated and processed efficiently [10]. This high assimilation efficiency generates more carrion-derived nutrients in the consumer versus detrital pool and also increases growth rates of foragers, buffering the ecosystem-wide capacity to support more predators and consequently their ability to control prey populations, which should in turn stabilize food webs [10], [61].

While the appearance of a whale carcass is largely a stochastic event, its importance to white sharks and the greater ecosystem should not be underestimated. Similar to the foraging behavior described in terrestrial apex predators (e.g. wolves, polar bears, spotted hyena), we suggest that white sharks scavenging on whales may be more prevalent and significant to the overall foraging ecology of the species, especially as individuals attain sexually mature size classes, and its role in shaping the social behavior, migrations, and community-wide impacts deserve further study.
